# Texture Analysis of Temporomandibular Joint Disc Changes Associated with Effusion Using Magnetic Resonance Images

**DOI:** 10.3390/dj12030082

**Published:** 2024-03-21

**Authors:** Camila Miorelli Girondi, Sérgio Lúcio Pereira de Castro Lopes, Celso Massahiro Ogawa, Paulo Henrique Braz-Silva, Andre Luiz Ferreira Costa

**Affiliations:** 1 Department of Stomatology, School of Dentistry, University of São Paulo (USP), São Paulo 05508-220, SP, Brazil; camila.girondi@usp.br; 2 Department of Diagnosis and Surgery, São José dos Campos School of Dentistry, São Paulo State University (UNESP), São José dos Campos 12245-000, SP, Brazil; sergio.lopes@unesp.br; 3 Postgraduate Program in Dentistry, Cruzeiro do Sul University (UNICSUL), São Paulo 01506-000, SP, Brazil; celsomassahiro@gmail.com (C.M.O.); alfcosta@gmail.com (A.L.F.C.)

**Keywords:** texture analysis, computer-aided diagnosis, diagnostic imaging, MRI, TMJ, TMD

## Abstract

The purpose of this study was to identify changes in the temporomandibular joint disc affected by effusion by using texture analysis of magnetic resonance images (MRIs). Methods: A total of 223 images of the TMJ, 42 with joint effusion and 181 without, were analyzed. Three consecutive slices were then exported to MaZda software, in which two oval ROIs (one in the anterior band and another in the intermediate zone of the joint disc) were determined in each slice and eleven texture parameters were calculated by using a gray-level co-occurrence matrix. Spearman’s correlation coefficient test was used to assess the correlation between texture variables and to select variables for analysis. The Mann–Whitney test was used to compare the groups. Results: The significance level was set at 5%, with the results demonstrating that there was no high correlation between the parameter directions. It was possible to observe a trend between the average parameters, in which the group with effusion always had smaller values than the group without effusion, except for the parameter measuring the difference in entropy. Conclusion: The trend towards lower overall values for the texture parameters suggested a different behavior between TMJ discs affected by effusion and those not affected, indicating that there may be intrinsic changes.

## 1. Introduction

According to Gaa et al. [1] and Ito et al. [2], the articular disc has an important function in the joint. Anatomically, the articular disc appears as a biconcave oval structure allowing all components to fit together in resting position and during movements. Behzadi et al. [3] reported that the articular disc of healthy asymptomatic individuals in the sagittal section presents a thinner center (about 1.5 mm), with the thickness of the anterior portion being slightly wider (about 2 mm) and the posterior portion being thicker (about 3 mm).

The biomechanical properties of the articular disc were described in a previous study [4], showing that the chondrocytes present in the cartilage of the disc are slightly different from other types of cells. Moreover, the shape of the articular disc not only confers geometric suitability to the components of the temporomandibular joint (TMJ) but also helps distribute the load and neutralize shocks and functional and parafunctional stresses [4,5].

The epidemiology of TMDs in Brazil presents a comprehensive overview of the prevalence and contributing factors to these conditions within the country. A meta-analysis study has delved into the multifaceted nature of TMDs, exploring variables such as gender, age, and socio-economic status, highlighting a significant prevalence of TMDs in both men and women, with a higher occurrence observed among women [6]. 

Effusion is the term used by radiologists for a sign of hyperintensity seen within a joint on MRI [7]. Joint effusion can also be observed through MRI, whose images appear as a T2-weighted hypersignal, and is associated with patients with symptomatic intra-articular disorders [8], and it could be presented as one of the parameters for evaluating TMD in patients [9].

MRI studies of TMD and TMJ are gaining popularity and have been described at different tesla levels, from 1.5 and 3 T to 7 T. The tesla level in MRI refers to the strength of the magnetic field used in the MRI machine. Tesla (T) is the unit of measurement for the magnetic field. The higher the tesla number, the stronger the magnetic field. Therefore, 1.5 T is the magnetic field most commonly used in conventional clinical MRI machines; 3 T offers a stronger magnetic field than 1.5 T devices. This can result in higher resolution and quality images in certain types of exams, such as functional MRI (fMRI) and musculoskeletal examinations, and 7 T devices are mainly used in research environments due to their strong magnetic field. They offer exceptionally high spatial resolution, enabling detailed imaging of anatomical structures and biological processes on a microscopic scale. However, they are less common in clinical settings due to concerns about safety and cost [10,11,12,13].

The effusion on MRI is observed usually showing disc displacements in advanced stages and is identified as an area of T2-weighted high signal intensity in the region of joint space [14]. It is characterized as the absence of effusion when no area showing a high signal is found or when there is only the presence of a line of high signal along the articular surface [15].

The inability to demonstrate articular disc perforations is a limitation of MRI [16,17]. However, it is known that when images demonstrate a reduction in joint space caused by the contact between the bones that comprise the TMJ, these images are associated with degenerative joint disease and are indirect signs that there is perforation of the articular disc that can be observed on T2-weighted images, including joint effusion [15]. Roh et al. [7] suggest that TMJ effusion may be related to disc displacement and degenerative changes (e.g., osteophytes, avascular necrosis, osteochondritis dissecans) and thus a marker of degeneration of TMJ.

Texture analysis (TA) is based on a sequence of algorithms used to characterize different types of spatial image variation, which are not always visible to the human eye. There are several approaches for extracting TA data from an image, with the gray-level co-occurrence matrix (GLCM) being one of the most used statistical approaches. GLCM is based on statistical measures related to the distribution of gray levels in the image, where each element (i, j) of the matrix corresponds to the number of times the gray-level “i” co-occurs with the gray-level “j” for a given distance and direction between pixels (for example, distances from 1 to 5 pixels and directions of 0°, 45°, 90°, and 135° are commonly used) [18].

TA is an imaging technique providing a quantitative means of extracting image features useful for comparative analysis as it has been reported to improve diagnostic accuracy and prognostic prediction [19,20]. TA can also be used to develop computational models by using advanced artificial intelligence algorithms as a tool for diagnosis and accurate treatment [21]. One study showed that two of the texture parameters (i.e., contrast and entropy) can identify changes in the functional status of masticatory muscles in patients with TMD, thus being considered an effective imaging biomarker for the evaluation of functional changes [22]. Therefore, the purpose of the present study was to evaluate whether MRI TA could detect potential changes in the articular discs of TMJ attributable to joint effusion.

Based on the premise that TA in MRI can provide detailed information about the composition and structure of tissues, it is determined that TA can serve as a tool to identify tissue changes. The articular disc and the textural characteristics in MR images can reveal specific patterns associated with effusion in the articular disc. Therefore, it is expected that TA can provide biomarkers and practical imaging to identify these changes and correlate them with relevant clinical findings on MRI in patients with articular disc dysfunctions.

## 2. Materials and Methods

### 2.1. Subjects

This is a retrospective study approved by the Research Ethics Committee of the School of Dentistry of the University of São Paulo (USP) according to protocol number 56631222.9.0000.0075. All subjects signed an informed consent form in accordance with the Declaration of Helsinki.

The sample size calculation was performed using G*Power 3.1.9.2 software (Düsseldorf, Germany). With 446 TMJs analyzed, encompassing 88 males and 135 females, our study surpasses the minimum recommended sample size of 142. In this study, the product-moment method was applied, a method for calculating effect size. The method was chosen because it is recommended when applying the Mann–Whitney test, a method for comparing groups while ensuring a confidence level of 0.95 and α = 0.05. The mean age of the subjects was 34.8 years old. This retrospective study evaluated MRI scans acquired from January 2019 to December 2020.

Eleven (11) texture parameters were measured in 12 different directions, totaling 132 variables. Because such a high number of variables and the resulting high number of statistical tests can lead to an error increase in the final analysis, the total number of variables is reduced. 

The articular disc is located between the mandibular condyle and the temporal bone, allowing translation and rotation movements, as well as mandibular movements. It is composed of fibrocartilage and its extracellular matrix consists of type I collagen. Articular disc: the anterior border contains significantly fewer collagen fibers than the anterior border and the central region, which have little or no metallurgical fibers [23]. On the posterior edge, as it is a fixation region, there is a greater density of these metallic fibers [17]. Due to this fibrous density, characteristic of the tissue in the posterior region of the articular disc, in MRI exams the region presents a higher image density, which could influence the tons of gray in the matrix chosen for the study. For this reason, the posterior band of the disc articular was not analyzed in the study. 

### 2.2. MRI

The MRI scans were acquired by using a 1.5 T scanner (Magnetom Essenza, SIEMENS, Munich, Germany) with a bilateral surface coil of 20 cm diameter. The parameters of the proton density sequence in the parasagittal plane were as follows: thickness (TH) of 2.5 mm, matrix of 512 × 512, spin echo (SE) with TE of 24 ms, TR of 2000 ms, and flip angle of 150°. The parameters of the T2-weighted sagittal sequence were as follows: thickness (TH) of 2.5 mm, matrix 512 × 512, and spin echo (SE) with TE of 83 ms, TR of 2180 ms, and flip angle of 150°. The parameters for mouth-open and mouth-closed positions were the same. 

The 1.5T MRI, a widely accepted and clinically established standard, provides a satisfactory compromise between spatial resolution and practical considerations. This field strength is recognized for its ability to capture detailed TMJ anatomy and pathology while remaining commonly available and more cost-efficient than higher tesla options. Therefore, our decision to employ a 1.5T MRI is justified, aiming to strike a balance between methodological rigor, clinical relevance, and resource optimization in the context of our research objectives.

### 2.3. Image Analysis

Analysis and interpretation of the MRI were performed on a consensual basis by two radiologists, who searched for pathological changes bilaterally [24]. Morphological disc changes were analyzed in proton density slices in the sagittal plane [7,25]. The presence of effusion was identified as an area of high signal intensity in the region of the superior and inferior joint spaces on T2-weighted images [26].

Thirty-six exams were also excluded in which images showed poor visualization of details and low clarity or condylar bone changes and discs with irregular morphology.

### 2.4. Image Processing 

The resulting data were converted into DICOM format for analysis with OnDemand3D^®^ software (version 1.0.9.3223 CyberMed Inc., Seoul, Republic of Korea) on a 23.8″ LED monitor (Dell-Ultrasharp, Austin, TX, USA), in which the oblique sagittal slice was chosen for each scan. Three consecutive slices were selected from the central area of TMJ. Next, these sagittal sections were saved in bitmap (BMP) format.

All these images in BMP format were exported to MaZda software (version 4.6, Technical University of Lodz, Institute of Electronics, Poland) for automated calculation of texture parameters.

**Figure 1:**
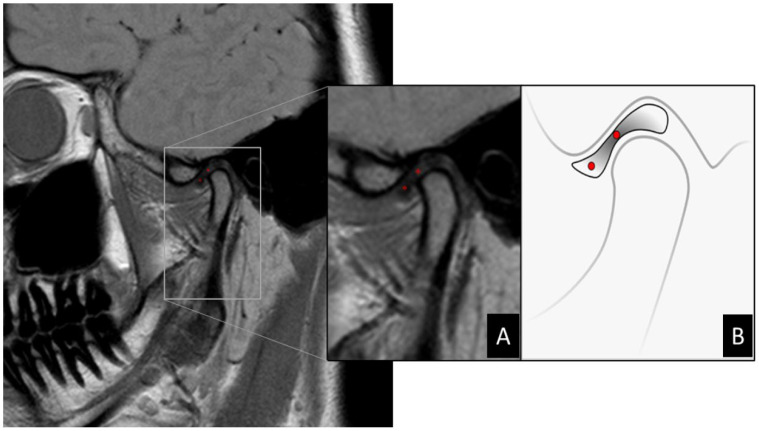
(**A**) Zoom in MRI image of TMJ demonstrating selection of ROI demarcated in red dots via MaZda software. (**B**) Illustration demonstrating the location of ROIs on MR image of the articular disc. Articular disc showing a biconcave shape divided into three parts: central thinner band, thicker posterior band, and anterior band. When normally positioned, the central band corresponds to the narrowest interosseous distance between the condyle and articular eminence. In this scheme, the two ROIs can be located in the central region of the anterior band and central band, respectively.

A round-shaped region of interest (ROI) of 44 pixels in diameter was manually traced in two positions for analysis, as shown in Figure 1. The posterior band of the articular disc was excluded from the study because it is an insertion region with the highest density of elastic fibers, which, although present in all regions, appear in greater numbers in the boundaries of the disc. The anterior edge of the disc contains significantly fewer fibers than the posterior edge and the central region has few or no elastic fibers [27]. The tissue and molecular characteristics of the region directly influence the appearance of the disc on MRI. Physiologically, the posterior edge of the disc shows hyperintensity in its central area due to the presence of transversely oriented collagen fibers. This apparent hyperdensity in the posterior border can affect the diagnosis of disc displacement, especially the partial ones [28].

### 2.5. Texture Analysis

Eleven texture parameters were extracted from each slice for each ROI, as can be seen in Table 1 were describes the parameters used for texture analysis in relation to grayscale related to neighboring pixels. Each parameter characterizes image properties such as roughness, contrast, homogeneity, and texture complexity. With them, it is possible to evaluate the average level of gray ROIs and the variation in their pixel values, showing the most significant parameters for discriminating between pathological and healthy tissues. TA statistics performs calculations on local characteristics simultaneously at every point in a texture image and obtains a collection of data from the distributions of these local characteristics [16].

The local characteristic is determined by the arrangement of intensities at specific positions in relation to each point in the image. Depending on the number of points used to determine the local characteristic, the statistics can be categorized as first-order, second-order, or higher-order statistics. The statistics provide diverse evaluations of texture attributes [16].

From a statistical point of view, the analyses should be started from two hypotheses: null and alternative ones. In the case of the present study, we can define the following:

- Null hypothesis: it is not possible to differentiate between texture parameter(s) of images of the articular disc extracted from MRI slices of patients with effusion and those without it.
- Alternative hypothesis: it is possible to differentiate between texture parameter(s) of images of the articular disc extracted from the MRI slices of patients with effusion and those without it.

### 2.6. Statistical Analysis

Spearman’s correlation coefficient was used to evaluate the correlation between texture variables and select variables for analysis. The Shapiro–Wilk normality test was applied. To compare the groups, the Mann–Whitney test was used. The significance level adopted was 5%. 

Data were analyzed by using the R software, version 4.2.0 (The R Foundation for Statistical Computing, Vienna, Austria).

## 3. Results

The sample data did not show a normal distribution. A paired t-test was performed to evaluate variations between textures found in the ROI for disc degeneration, whereas Spearman’s correlation coefficient test was used to assess the correlation between texture variables and selected variables for analysis. For comparison of the groups, the Mann–Whitney test was used. The significance level was set at 5%. Spearman’s correlation coefficient test was used to assess the correlation between texture variables and selected variables, whereas the Mann–Whitney test was used to compare the groups.

As the Spearman test is a statistical and non-parametric measure, it measures the correlation between two variables, the relationship between them can be described by some mathematical expression, that is, they do not have random dispersion and are classified ordinally. Thus, the closer the coefficient is to +1 or −1, the greater the relationship between the variables. On the other hand, the closer the coefficient is to zero, the more random the variables will be and the smaller the relationship between them will be. The sign indicates the upward or downward slope of the curve and the Mann–Whitney test compares the distribution between two different groups. This is a non-parametric test used to compare two independent samples and determine whether they are statistically different from each other. It is applied when the data do not follow a normal distribution or when the variables of interest are on the ordinal scale. It compares the medians of the two samples to assess whether they are equal or different.

The significance level was set at 5%, that is, this is the limit to reject or not the null hypothesis based on the *p*-value.

The graphs demonstrated that there is no high correlation between some directions regarding the parameters Contrast, Correlat, InvDfMom, DifVarnc, and DifEntrp, meaning that it is not possible to choose only one direction for these parameters to carry out the analyses and, consequently, two approaches were used as follows:

1. Average between all directions for each of the parameters;
2. Direction S(1,0).

**Figure 2:**
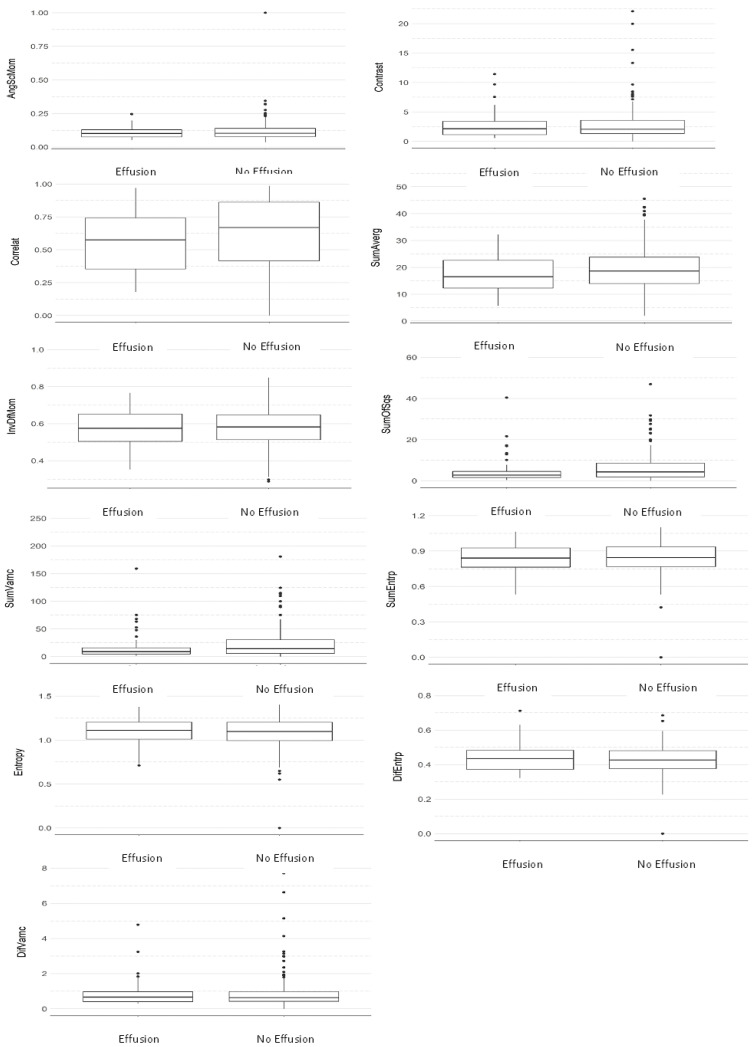
Comparative boxplot between groups considering the average of all directions (Mann–Whitney test).

**Figure 3:**
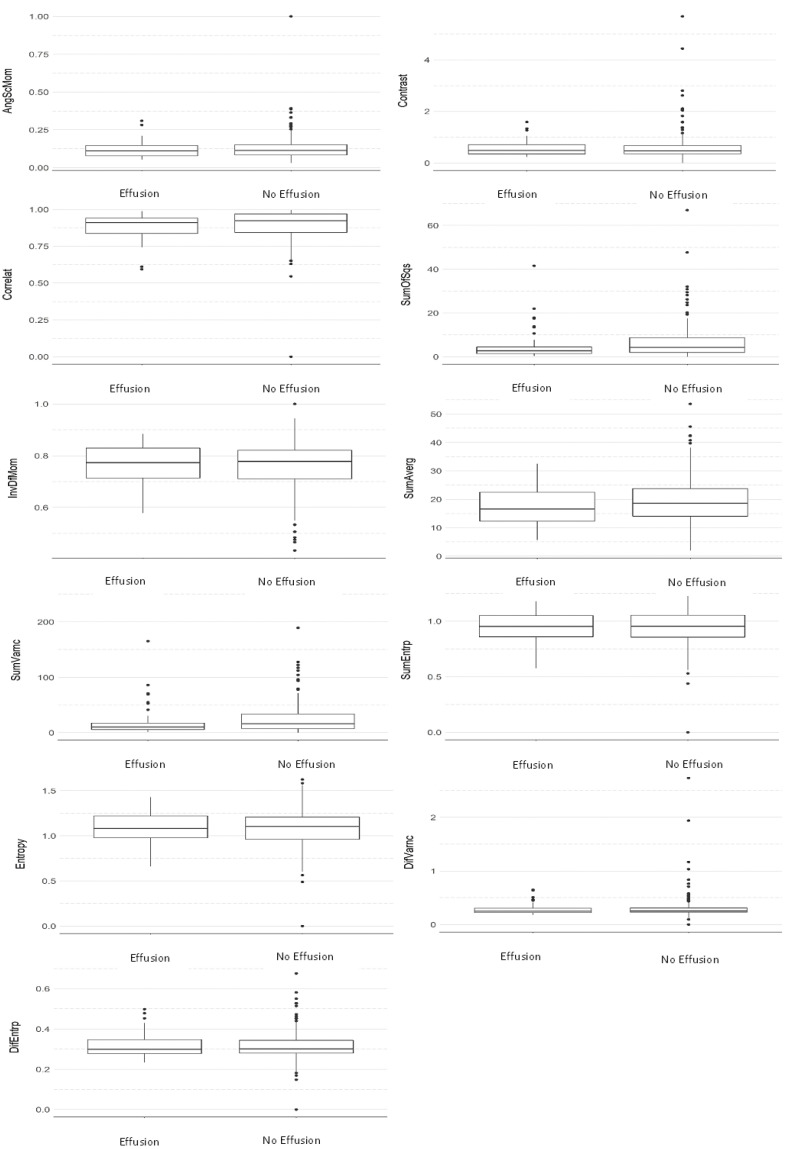
Comparative boxplot between groups considering the S(1,0) direction (Mann–Whitney test).

Table 2 and Table 3 and Figure 2 and Figure 3 show that no statistically significant differences were found between the two groups in any of the texture parameters when considering the average of all directions (Table 2) (Figure 2) and direction S(1,0) only (Table 3) (Figure 3). In addition, it can be seen via the effect size parameter that all texture parameters presented a low magnitude of effect on both tables. Effect size values between 0.1 and 0.3 were considered to have a small effect, between 0.3 and 0.5 a medium effect, and greater than 0.5 a large effect, in accordance with Cohen [30].

## 4. Discussion

For analysis of soft tissues in the TMJ, MRI is the best method [31,32,33,34] because it not only provides accurate images and three-dimensional sections but also allows us to visualize relationships with adjacent structures and their functioning [35].

The effusion is suggested as an inflammatory process in imaging exams. On T2-weighted MRI images, effusion manifests itself as areas of high signal in the joint space [36]. Other studies found that effusion is common in temporomandibular disorders, being present in up to half of the joints with some degree of disc displacement [7,37], which results in the release of inflammatory mediators [38].

Imaging diagnostic methods depend on the visual assessment performed by a radiologist so that the joint and its attachments can be visualized, which involves a direct reference to the spatial arrangement and morphology of its components [39]. For this reason, the images were independently evaluated by two radiologists in the present study in order to include only those positively diagnosed cases of effusion on a consensual basis. Patients who have effusion usually present some degree of damage to the articular disc, which can lead to inflammation capable of generating an accumulation of fluid inside the joint and, in some cases, stretching or displacement of the articular disc, thus further aggravating the condition.

However, it is often not enough to analyze the properties of an image based only on the visualization. Moreover, several characteristics and properties of the tissue in question should be taken into account [40].

For this reason, the proposition of new techniques for diagnostic imaging has been developed, such as the AT technique, which is capable of quantifying image heterogeneities that may not be appreciated with the naked eye and represents a method based on mathematical analysis to assess the intensity of the gray level and the position of the pixels in the images, providing “texture features”, which represent a quantitative measure of various imaging techniques [16]. TA typically involves several steps including image segmentation, feature extraction, and statistical analysis. Image segmentation is the process of separating the area of interest (the articular disc) from the surrounding tissues and structures, while feature extraction involves identifying specific texture features (the evaluated parameters). In this work, the eleven image texture parameters are listed in Table 1.

Statistical analysis is then used to quantify differences in these characteristics between different disc regions and between different images acquired. By analyzing diagnostic patterns for the articular disc over time, researchers can gain valuable information about the progression of disc degeneration and other pathologies [16]. It was suggested that diagnostic imaging based on automated tools was able to accurately validate the diagnosis of osteoarthritis in 2D and 3D images, but in comparison, they performed poorly in detecting articular disc disorders from MRI data. Moreover, variables usually affecting analyses of inflammatory changes in joint and disc disorders are the following: complexities in the clinical classification criteria, non-standard diagnostic parameters, and errors in image acquisition [36].

In order to extract information about the properties of the tissue, it is necessary to describe the characteristics (parameters) to quantify the textures and thus achieve the necessary precision. Texture parameters defined as numerical values can be applied to classify different regions of the image.

According to Ramola et al. [20], GLCM is the most efficient method for extracting texture features for classification and discrimination purposes.

Based on the results described, it can be seen that the average imaging parameters of MRI of patients with effusion are always lower than that of those without it, except for the parameter difference of entropy (Table 2). Still, from a statistical perspective, it is common to say that when the *p*-value is lower than 0.1 it tends to be significant, but in order to reach the statistical significance, *p* < 0.05. In the present study, Table 2 shows the parameters sum of squares, which represents a measure of dispersion (related to the average) of shades of gray (*p*-value = 0.067, effect size 0.12—considered a small effect), and sum of variances, which represents the dispersion of the average sum of grayscale distribution (*p*-value = 0.06, effect size 0.13—considered a small effect). In Table 3, the parameters sum of squares and sum of variances presented *p*-values of 0.07, effect size 0.12—considered a small effect and 0.075, effect size 0.12—considered a small effect, respectively.

In addition, Table 2 also considered a small effect on the parameters: correlation—effect size 0.10, sum of means—effect size 0.10. Additionally, in Table 3 as well as considering a small effect, the following were found: sum of average—effect size—0.10, sum of variances effect size—0.12.

Furthermore, it can be seen that the greatest difference is found in the parameter sum of variances as its value for patients with effusion is 21.7% lower than for those without it. By comparing their averages, it can also be seen that this parameter tended to be above the average, i.e., that the magnitude of the sum of variances also tends to be high in patients with effusion.

However, in agreement with the study by Juras et al. [41], some texture features extracted from quantitative MRI maps correlate with the stage of cartilage degeneration evaluated histologically; thus, it is worth highlighting that with the presence of several outliers (i.e., samples with very discrepant values) for all parameters extracted from MRI of patients with effusion, the mean becomes a statistical parameter not suitable for characterizing imaging parameters in this group of patients with effusion. However, other statistical parameters should be used to characterize the image parameters in the sample group used in this study.

Regarding the level of sample dispersion for each parameter (measured here through standard deviation), it can be observed that all imaging parameters of patients with effusion showed greater regularity compared to those of patients with no effusion. Thus, we can say that among the patients evaluated in the present study, those with effusion tended to present more regular texture (i.e., gray-level patterns) in the MRI slices of the articular disc (i.e., ROI with less variation).

Despite these results, it is not possible to establish a limitation for texture parameters as several of these tended to show statistical differences in cases in which joint effusion and bone marrow edema could not be evaluated, even if they could have been present. On the other hand, other studies could be carried out by expanding the sample for comparison with our findings.

## 5. Conclusions

The trend towards lower overall values for the texture parameters analyzed shows a different behavior between TMJ discs affected by effusion and those not affected, indicating that there may be intrinsic alterations that are not perceptible by only using visual analysis of the MRI.

## Figures and Tables

**Table 1:** Texture parameters extracted in the analysis [29].

Texture Parameter	Abbreviation	Description
Angular second moment	AngScMom	Measurement of image uniformity
Contrast	Contrast	Represents the amount of local variation in gray level
Correlation	Correlat	Linear measure dependence of gray level between neighboring pixels
Sum of squares	SumOfSqs	Measurement of the dispersion (related to average) of gray-level distribution
Inverse difference moment	InvDfMom	Homogeneity of the distribution of gray level on the image
Sum of average	SumAverg	Mean of the distribution of the sum of gray-level values
Sum of variance	SumVarnc	Dispersion around the mean of the sum distribution of gray level
Sum of entropy	SumEntrp	Disorganization of the sum distribution of gray level
Entropy	Entropy	Degree of disorder between pixels in the image
Difference of variance	DifVarnc	Dispersion of the gray-level difference
Difference of entropy	DifEntrp	Disorganization of the gray-level difference

**Table 2:** Difference between groups considering the average of all directions and the values close to statistical significance.

Parameter	Group	Average	Maximum	*p*-Value	Effect Size
AngScMom	Effusion	0.111	0.247	0.604	0.03
	No effusion	0.121	1.000		
Contrast	Effusion	2.820	11.400	0.891	0.01
	No effusion	2.950	22.100		
Correlat	Effusion	0.560	0.970	0.102	0.11
	No effusion	0.625	0.985		
SumOfSqs	Effusion	5.43	40.50	0.067	0.12
	No effusion	6.91	65.80		
InvDfMom	Effusion	0.574	0.767	0.725	0.02
	No effusion	0.580	1.000		
SumAverg	Effusion	17.4	32.2	0.137	0.10
	No effusion	19.7	53.5		
SumVarnc	Effusion	18.9	159.0	0.06	0.13
	No effusion	24.7	250.0		
SumEntrp	Effusion	0.839	1.060	0.822	0.02
	No effusion	0.841	1.170		
Entropy	Effusion	1.100	1.380	0.729	0.02
	No effusion	1.090	1.520		
DifVarnc	Effusion	0.903	4.780	0.995	0.00
	No effusion	0.920	7.700		
DifEntrp	Effusion	0.441	0.712	0.816	0.02
	No effusion	0.434	0.787		

**Table 3:** Difference between groups considering only the direction S(1,0) and the values close to statistical significance.

Parameter	Group	Average	*p*-Value	Effect Size
AngScMom	Effusion	0.122	0.678	0.03
	No effusion	0.132		
Contrast	Effusion	0.572	0.981	0.00
	No effusion	0.627		
Correlat	Effusion	0.883	0.205	0.09
	No effusion	0.893		
SumOfSqs	Effusion	5.47	0.070	0.12
	No effusion	6.95		
InvDfMom	Effusion	0.762	0.848	0.01
	No effusion	0.763		
SumAverg	Effusion	17.4	0.139	0.10
	No effusion	19.8		
SumVarnc	Effusion	21.3	0.075	0.12
	No effusion	27.2		
SumEntrp	Effusion	0.939	0.867	0.01
	No effusion	0.939		
Entropy	Effusion	1.090	0.963	0.00
	No effusion	1.090		
DifVarnc	Effusion	0.291	0.982	0.00
	No effusion	0.311		
DifEntrp	Effusion	0.321	0.969	0.00
	No effusion	0.318		

## Data Availability

The datasets generated and/or analyzed during the current study are available from the corresponding author upon reasonable request.
